# Tension band suture in isolated tibial tubercle avulsion: A case report and review literatures

**DOI:** 10.1016/j.ijscr.2020.04.029

**Published:** 2020-05-08

**Authors:** Wachiraphan Parinyakhup, Tanarat Boonriong

**Affiliations:** Department of Orthopaedic Surgery and Physical Medicine, Faculty of Medicine, Prince of Songkla University, 15 Karnjanavanich Road, Hat Yai, Songkhla, 90112, Thailand

**Keywords:** Tibial tubercle avulsion, Tension band suture

## Abstract

**Introduction:**

Isolated tibial tubercle avulsion in adults are extremely rare, with only a few cases having been reported. The common treatment is screw fixation, followed by immobilization. We report a rare case of an isolated tibial tubercle avulsion in an adult and demonstrate the results of a tension band suture without post-operative immobilization for treatment.

**Case presentation:**

A 64 year-old woman presented with an isolated tibial tubercle avulsion, and loss of her extensor mechanism. Treatment with tension band suture could provide early motion, and her return to full ambulation in 2 months.

**Conclusion:**

An isolated tibial tubercle avulsion, there are many surgical techniques to secure the fixation. The tension band suture is one technique that the patient can acheive a good fixation without post-operative immobilization and rapidly return to normal activity.

## Highlights

• The incidence of isolated tibial tubercle avulsion is rare.
• After fixation of avulsion tibial tubercle by tension band suture, the patient can early motion of the knee.
• This surgical technique can use either simple avulsion tibial tubercle or comminuted avulsion tibial tubercle.

## 1 Introduction

Isolated tibial tubercle avulsion in an adult are extremely rare, with only a few reported cases having been reported [1,2]. This type of fracture is common in children and adolescents, but very rare in adults. In adolescents the mechanisms of injury are due to violent contraction of the quadriceps during extension (jumping), or by acute passive flexion of the knee against contracting quadriceps(landing) [3]. The mechanisms of injury in adults are direct trauma with eccentric contraction of the quadriceps [1]. Most of the reported cases were treated with screw fixation, and post-op immobilization; such, as a long leg cast or knee bracing for 4–6 weeks.

The purpose of this report is to present a rare case of an isolated tibial tubercle avulsion in an adult, which was treated with fixation of the fracture using a tension band suture, the early post-operative range of motion, and the results of treatment. This method of fixation can also be applied for tibial tubercle fractures, combined with tibial plateau fractures and tibial tubercle avulsion in adolescents. This case report was made according to the SCARE criteria [4].

## 2 Presentation of case

**Fig. 1:**
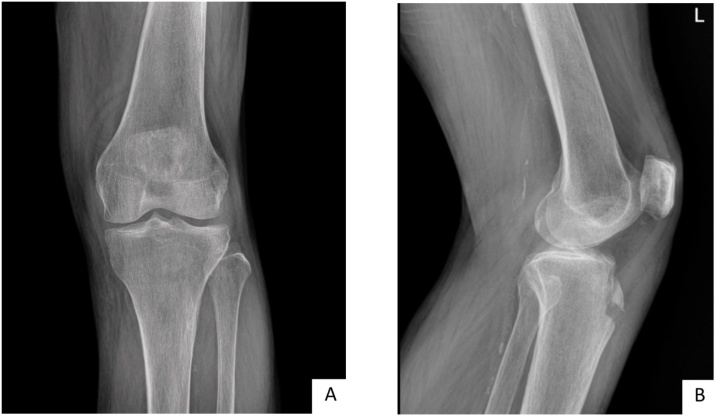
The initial radiographic imaging, (A) anteroposterior view of left knee, (B) lateral view of right knee.

A 64-year-old female presented with right anterior knee pain after a simple fall. She reported her right knee hit the floor from a kneeling position. She was unable to bear-weight- and actively extend the knee. On examination she had swelling and tenderness anterior to tibial tubercle, without joint line tenderness or joint effusion. She was unable to actively extend her knee and could not do a straight leg raise. She had a medical history of hypertension, and type II diabetes. She was a housewife and was previously mobilized independently. A radiographic examination showed the tibial tubercle avulsion (Fig. 1A, B).

## 3 Treatment

**Fig. 2:**
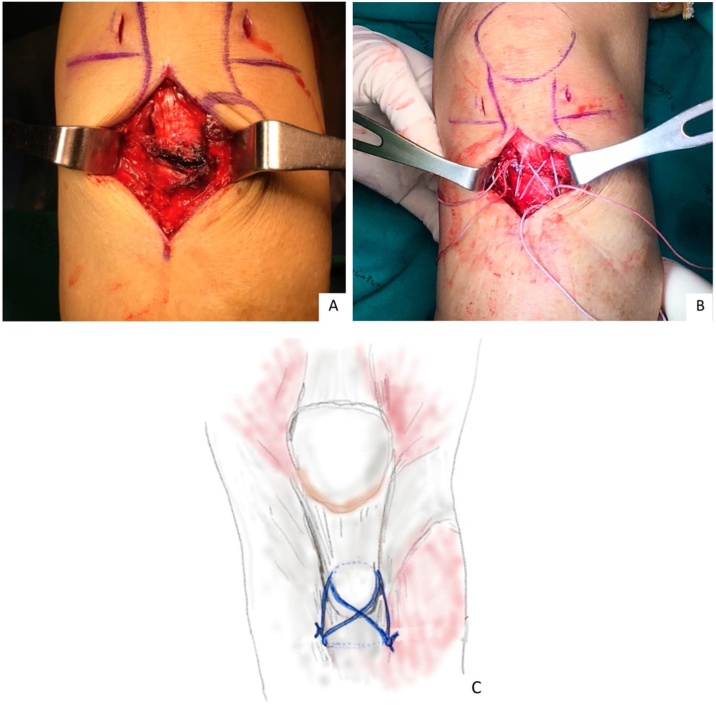
Intraoperative of avulsion of left tibial tubercle, (A) before tension band suture, (B) after tension band suture, (C) the illustration image of tension band suture technique.

Initial management was application of a posterior knee slap in a slightly flexion position. Operative treatment was started at 2 day after the injury. The operation started with an arthroscopic diagnosis, to rule out an intra-articular lesion, and we found minimal hemarthrosis without intra-articular structure injury. After arthroscopic diagnosis, the straight vertical medial para-patellar incision, 5 cm in length, was made from the joint line to a few centimeters distal to the fracture site. The medial and lateral soft tissue flaps were developed to be just superficial to the fracture site. The tibial tubercle was identified, and the epithenon was incised for clear vision of the fracture site as well as the patellar tendon (Fig. 2A). The fracture bed was carefully cleared of debris, in order to remove fracture hematoma. A 4.5 mm reamer (Smith and Nephew®) was used to create a bone tunnel, few centimeters distally from the fracture. Two No.5 FiberWires (Arthrex®) sutures was placed posteriorly to the patellar tendon, which created a figure-of-eight and a loop configuration. The fracture was reduced when tension was applied to the FiberWire (Fig. 2B, C). The stability of fixation was tested, before knot tying, and we found stable fracture reduction along with a full passive range of motion

**Fig. 3:**
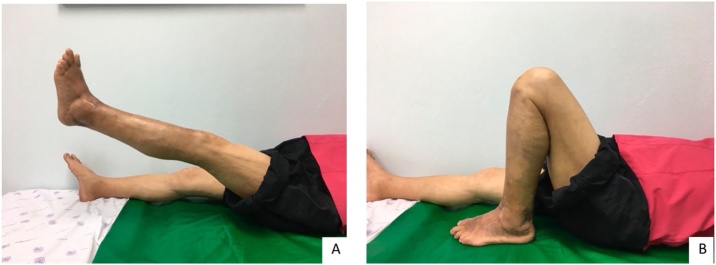
The range of motion of left knee in (A) extension and (B) flexion at 4-week follow up.

**Fig. 4:**
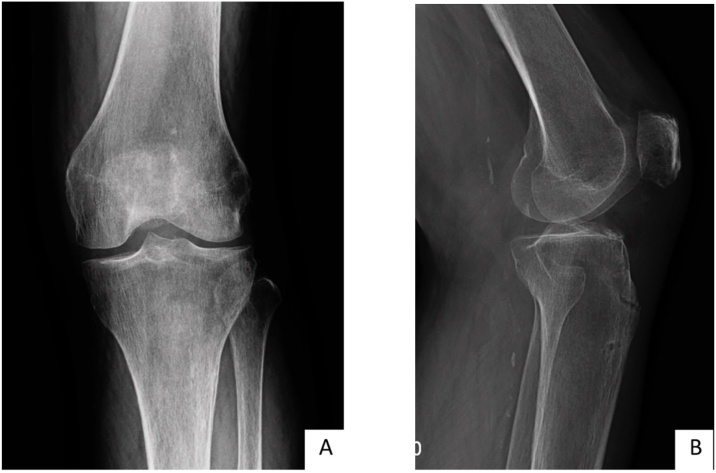
The radiographic imaging in (A) anteroposterior view, (B) lateral view of left knee at 3-months post-op.

Postoperatively, the knee was allowed to have an early range of motion, from 0 to 60 degrees, coupled with partial weight bearing using a walker. A 0–60 degrees ROM knee brace was used for the first 2 weeks, and a full ROM at 4 weeks. The patient was evaluated at 2, 4, 6 weeks and 12 weeks. At 4 weeks, post op, the patient was pain free, had nearly full range of motion, and full active extension of the knee was achieved (Fig. 3A, B). The radiographic showed healing at 3 months post-op (Fig. 4A, B).

## 4 Discussion

Acute tibial tubercle avulsion fractures are uncommon. These injuries typically occur in adolescent boys involved in certain sports. These fractures were classified according to Ogden’s classification (Table 1) [5].

**Table 1:** Ogden’s classification of injury to tibial tubercle.

Type	Pattern
Type 1A	Fracture line leads through the ossification center of the tubercle without displacement
Type 1B	The fragment is displaced anteriorly and proximally
Type 2A	Fracture line leads through the junction of the ossification of the proximal end of the tibia and the tubercle
Type 2B	The tubercle fragment is comminuted
Type 3A	Fracture line extends to the joint and is associated with discontinuity of the joint surface
Type 3B	The tubercle fragment is comminuted

The mechanism of injury is usually an indirect force caused by sudden contraction of the quadriceps muscle. During take-off for a jump, the quadriceps mechanism forcefully contracts against the patellar tendon insertion. When the force exceeds the strength of the tibial tubercle physis, a fracture is generated, leading to avulsion of the tibial tubercle. Acute passive flexion of the knee against contracting quadriceps, or eccentric contraction is another mechanism of injury [1,3,6]. The common treatment was closed, or open reduction and internal fixation followed with immobilization for 4–6 weeks [1,3,[6], [7], [8]], and results of treatment are excellent. Another choice of treatment is screw fixation, and tension band wiring with patellar, without post-op immobilization, but removal of the tension band wiring being required [2].

Tibial tubercle avulsion fractures in adults are mostly related to high energy trauma, and are correlated with associated injuries around the knee (femoral shaft fractures, intra-articular fractures and tibial shaft fractures) [[9], [10], [11]]. An isolated tibial tubercle avulsion fracture in adults, without predisposing factors is extremely rare. Only a few case reports are reported in literatures [1,2]. Two of them were of an elderly age, and were related to simple falls, involving a direct hit to the anterior knee in the knee flexion position, one was like Ogden type IB and Type IIIA. Our case was a 64-year-old female suffering from a simple fall coupled with a direct hit to the anterior knee. According to the Ogden classification our case was type IB.

**Fig. 5:**
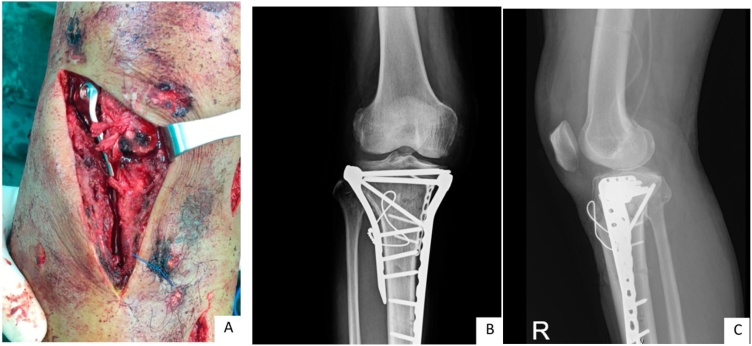
The clinical imaging (A) and radiographic imaging in (B) anteroposterior view, (C) lateral view of right knee.

The AO Foundation describes three methods for tibial tuberosity fracture treatment. The first method involves non-operative treatment with casting or bracing in non-displaced and stable fracture. The second method involves fixation by lag screws with or without tension band wiring as an augment fixation. The third method involves anchor suture. If there is any doubt as to the security of the fixation, the limb should be immobilized in a cylinder cast for six weeks (Fig. 5).

There is controversy between keeping the knee immobilized, and the postoperative immobilization period. Isolated tibial tubercle avulsion was found in elderly group, whereas most of these consisted of small fragments. The principle of treatment was acceptable reduction and a strong fixation that allows early motion. Our case used tension band suturing with No5 FiberWire and created two loops of tension band, a figure-of-eight fashion for compression, and a cerclage fashion to prevent rotational force. The tension band systems will neutralize tension force at the anterior cortex, allowing for compression force at the fracture site. This type of fixation is strong enough for early activity along with a passive range of motion, allowed for early post-operative motion, and not necessary to remove the instruments such as the tension band wiring.

Screw fixations of a tibial tubercle fracture in adolescents has had reported complications, for example: recurvatum deformity from physeal injury and growth arrest [12,13], vascular injury to popliteal artery as it passes over the distal metaphyseal fragment, and loss of motion from arthrofibrosis, due to long time immobilization [14]. The Tension band systems has many advantages in the treatment of tibial avulsion in adolescents; such as, no risk of physeal plate injury, no need for fluoroscopy in the operating room as well as allowing for early range of motion. In researcher opinions this method of fixation may be applied to Ogden type IB, IIA and IIB tibial tuberosity fractures.

The tibial tubercle fracture can also be found in high energy trauma cases, and is associated with fractures around the knee [9,10,15]. Maroto and Dunbar reported incidence of tibial tubercle fractures as 16 % in 127 cases of tibial plateau fracture [15]. Common treatment of tibial tubercle fractures, in tibial plateau fractures, were screws or plate fixation with post-op immobilization. The tension band system can be used for fixation of tibial tubercle fractures, in tibial plateau fractures, allowing early ROM exercise, and can also be used for fixation in cases of comminuted fractures of tibial tubercle, which are difficult to fix with screws.

After the plateau fixation with plates and screws, a tension band wiring was applied, which allowed for early post-op motion.

In our opinion the proper diagnosis of the injury, stable fixation and early motion are the principles of the fracture treatment. This method provides a stable fixation and allows early motion in patients with isolated tibial tubercle fractures, with excellent knee function at 3 months post operation. This method can also be used for tibial tubercle fractures in complex tibial plateau fractures, where fixation of such comminuted tibial tubercle fractures is very difficult.

## 5 Conclusion

An insolated tibial tubercle avulsion, there are many surgical techniques to secure the fixation. The tension band suture is one technique that the patient can acheive a good fixation and rapidly return to normal activity.

## Declaration of Competing Interest

No conflicts of interest.

## Funding

No funding was involved regarding this case report.

## Ethical approval

The present study was approved by the Prince of Songkla University Institutional Review Board, Faculty of Medicine, Songklanagarind Hospital, Prince of Songkla University.

## Consent

Written informed consent was obtained from the patient for publication of this case report and accompanying images. A copy of the written consent is available for review by the Editor-in-Chief of this journal on request.

## Author contribution

Wachiraphan Parinyakhup—Preparation of case report, Literature review, Writing the paper.

Tanarat Boonriong—Preparation of case report. Writing the paper.

## Registration of research studies

None.

## Guarantor

Wachiraphan Parinyakhup, MD.

## Provenance and peer review

Not commissioned, externally peer-reviewed.
